# Note from the Editors: Global Theme Issue

**Published:** 2007-10

**Authors:** Kenneth S. Korach, Steven R. Kleeberger, Matthew P. Longnecker

**Affiliations:** *EHP*, E-mail: EHPEditor@niehs.nih.gov; Interim Deputy Editors, *EHP*

For the last few years, *Environmental Health Perspectives* (*EHP*) has been involved in international efforts, including publication of a quarterly Chinese-language edition beginning in 2001, participating in the African Journal Partnership (working with *Mali Médical* since 2004), and contributing material to the journals *Ciência & Saúde Coletiva* (in Portuguese) and *Ciencia y Trabajo* (in Spanish).

According to the Council of Science Editors (CSE 2007a), journals seeking to promote scientific and medical research and publication in developing countries should

- Make content “globally relevant”
- Work with journals from developing countries to provide guidance and training
- Overcome the “global publishing gap” by assisting authors with language issues and publication, as well as experimental design
- Seek diversity in editorial boards, reviewers, and authors.

To further address the “global publishing gap,” the CSE (2007a) has established a task force on AuthorAid, a program to assist authors, primarily in developing countries, in publishing articles in competitive journals (Freeman and Robbins 2006).

We are pleased to participate in the CSE Global Theme Issue on Poverty and Human Development, the goal of which is “to raise awareness, stimulate interest, and stimulate research into poverty and human development” (CSE 2007b). More than 200 journals from both developed and developing countries are participating in this endeavor (CSE 2007b).

Our contributions to this theme include this editorial, the Spheres of Influence article (p. A500), and articles by Chen et al. (p. 1415) and Hall et al. (p. 1503).
